# Hot spring bathing practices have a positive effect on mental health in Japan

**DOI:** 10.1016/j.heliyon.2023.e19631

**Published:** 2023-08-30

**Authors:** Midori Takeda, Hiroki Nakamura, Hajime Otsu, Koshi Mimori, Toyoki Maeda, Shunsuke Managi

**Affiliations:** a Urban Institute & Department of Civil Engineering, Kyushu University, Japan; b Institute of Social Science, The University of Tokyo, Japan; c Department of Surgery, Kyushu University Beppu Hospital, Japan; d Department of Cancer Biology and Genetics, Comprehensive Cancer Center, The Ohio State University, United States; e Department of Internal Medicine, Kyushu University Beppu Hospital, Japan

## Abstract

Hot springs have long been used for medical purposes throughout the world. Recently, the positive effects of hot spa-bathing on circulatory diseases have been reported, while there are few reports on the mental effects of hot spa-bathing. Therefore, the purpose of this study was to clarify the relationship between hot spa-bathing habits and mental health throughout Japan. We conducted a nationwide online survey, including questions on bathing behavior, subjective satisfaction, lifestyle, and illness. The results showed a significant positive correlation between hot spa-bathing habits and multiple subjective satisfaction levels regarding mental health effects. The factor analysis results indicated that hot spa-bathing habits tended to be associated with good mental health, high health consciousness, and disease. Our study revealed that subjective satisfaction was higher among individuals with hot spa-bathing habits, suggesting that the hot spring spa-bathing habit may have a positive influence on mental health.

## 1 Introduction

Health benefits are often associated with specific geographical locations or regions called therapeutic landscapes [[1], [2], [3], [4]]. Therapeutic landscapes demonstrate the importance of places for maintaining physical and mental health [4].

Balneotherapy, spa therapy, hot springs, and water-related healing landscapes also form a major subtheme within the studies of holistic medicines [2,5]. In balneotherapy, bathing, drinking or inhalation of minerals and thermal waters, peloids, and gases is used as treatment [6,7]. Spa therapy, which has often been used in the past in Europe and the Middle East, includes balneotherapy, exercise, and massage as complementary treatments [6]. Some studies, such as those by Neumann et al. (2001) and Van Tubergen et al. (2001) [[8], [9], [10]], have reported that spa therapy may have beneficial effects on sleep and quality of life for patients with musculoskeletal and dermatological diseases [11].

In Japan, there are thousands of high mountains, many of which have volcanic activity and hot springs [11]. Japanese hot spring bathing has a history of over 2000 years, and there are over 27,000 sources of hot springs in more than 3000 locations [2]. Due to this geographical peculiarity, Japanese people prefer hot spring bathing in addition to regular bathing. A unique traditional health practice called ‘*Touji’* has developed in which symptoms are remedied by hot springs.

Hot spring waters have traditionally and empirically been valued for their therapeutic properties based on their composition, mineral concentration, and temperature. As a result, different spas are recommended for various medical conditions related to the gastrointestinal tract, respiratory system, ear, nose, throat, skin, gynecology, and rheumatology [[12], [13], [14]]. Whether the water is high in sulfur, bicarbonate, sodium chloride, bicarbonate-chloride, or other mineral salts, all spas were recommended for rheumatological diseases and osteoarticular trauma [12,14]. The common goals of the therapies such as balneotherapy and spa therapy for different patients are to relieve pain, improve functional capacity and health-related quality of life, and increase awareness of their disease [12].

Regarding the Japanese situation, Maeda et al. (2018) [15] focused on the body-warming effect of hot spa bathing in the case of elderly Japanese residents of Beppu city, Oita Prefecture, which has the highest concentration of hot spa sources in the world [15]. They conducted a questionnaire survey regarding hot spa-bathing habits and disease history among 20,000 residents aged ≥65 years living in Beppu city. The results showed that habitual hot spa bathing exerts preventive or promotive effects on the occurrence of certain diseases, such as hypertension (preventive) and colon cancer survival (promotive), in men [15]. Sekine et al. (2006) [11] examined whether spa resort use is associated with the health of Japanese employees aged 20–65 and indicated that spa use may benefit physical and particularly mental health, although longitudinal research is necessary to clarify causality.

Although Kamioka et al. (2019) [16] pointed out that the relationship between the use of hot water spas and patients’ mental health should be clarified by well-designed cohort studies, they found that a lower frequency of hot water spa bathing was significantly associated with an increased risk of underlying diseases among middle-aged and elderly patients.

Based on the literature mentioned above, it is indispensable to clarify the overall relationship among various factors, such as health status, types of diseases, daily activities, spa bathing, and subjective preferences. Therefore, this study aimed to verify the association between hot spring bathing and mental health in Japan and understand the psychological effects of hot spa bathing as a habit.

## 2 Methods

### 2.1 Data collection

To investigate the relationship with mental health, lifestyle, and hot spa bathing, we have conducted a questionnaire survey in Japan. We collected the necessary data through an online survey company Rakuten Insight, Inc. (Tokyo, Japan). Rakuten Insight is a Japanese online survey site with over 2.2 million monitors, allowing any monitor to complete surveys via their online site. To construct a less biased sample, Rakuten Insight survey monitors were sorted by region of residence, sex, age, and zip code and then subjected to systematic sampling. The questionnaire survey was conducted from November 15 to 25, 2019, to answer the questions developed by the authors, and responses were collected and tabulated.

All the study was performed in accordance with the Declaration of Helsinki. Informed consent was obtained from study participants. Ethics Committee of Institute of Social Science, The University of Tokyo approved the questionnaire study (permission number: 100).

### 2.2 Questionnaire variables

The questionnaire included items related to hot spring spa-bathing habit, characteristics, disease, and subjective items such as wellbeing and health status (Supplementary Material). The variables assessed in the study are the followings: spa-bathing frequency, spa bath immersion duration, spa-bathing period, 14 types of diseases incidence, age, weight, height, life satisfaction, job satisfaction, health condition, job stress, health stress, free days, breakfast, vegetable intake, salt intake, meat intake, amount of food, walking habit, stairs use, smoking, alcohol. The responses to subjective items were rated on a 5-point Likert scale.

Supplementary Table S1-6 summarizes the definitions of the variables used in this nationwide questionnaire survey and the descriptive statistics. We collected the data of individuals regarding their age, sex, weight, height, hot spring-bathing frequency, hot spring immersion duration, and hot spring-bathing period (Supplementary Tables S1 and S2). Subjective satisfaction (life/job/health conditions) and strength of stress (job/health conditions) were self-reported using the criteria in Supplementary Tables S3 and S4. In addition, to identify the participants’ health consciousness preferences, we obtained lifestyle-related information on diet, exercise habits, smoking habits and drinking habits (Supplementary Tables S3 and S5). Individuals were also asked to answer the diseases they have had in the past and the prevalence were listed in Supplementary Table S6.

### 2.3 Statistical analysis

Statistical analyses were performed using STATA (MP version 16.1) and R software (version 4.2.1). Statistical significance was set at p < 0.05. For correlation plots, Spearman's rank correlation coefficient was used because of the inclusion of ordinal variables in the data. No normality test was performed due to the nonparametric nature of the method. Ordinal logistic regression was used for the regression analysis to examine the effect of hot spring spa bathing habits on life satisfaction.

## 3 Results

### 3.1 Data summary

**Fig. 1:**
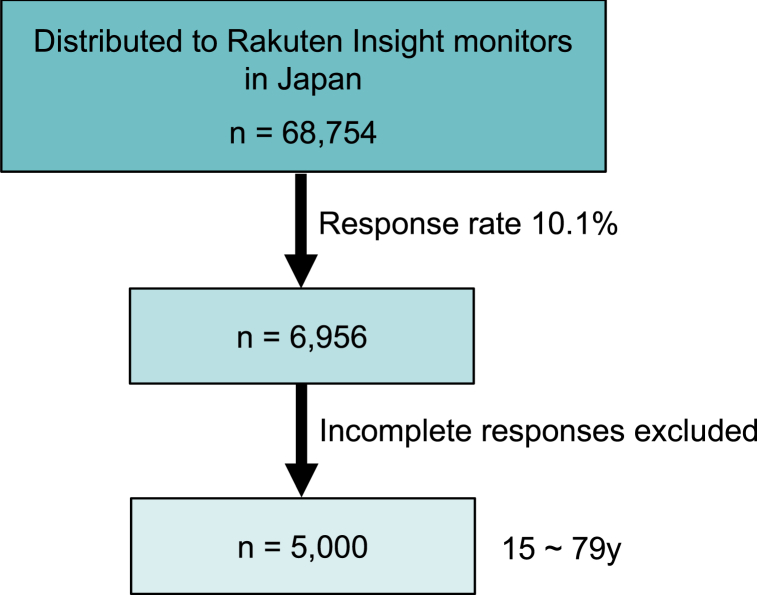
**Flowchart of the population selection process of the questionnaire study.** The selection flow for the study is indicated. The survey data were from all over Japan.

Rakuten Insight distributed a survey invitation email to 68,754 individuals and obtained 6956 responses (response rate: 10.12%). After excluding responses with incomplete answers, a final sample size of 5000 individuals (males: 2,485, females: 2515) was obtained (Fig. 1). Regarding the age distribution of the respondents, the numbers of respondents in their 20s or younger, in their 30s, 40s, 50s, 60s, and 70s or older were 923 (18.5%), 747 (14.9%), 942 (18.8%), 785 (15.7%), 883 (17.7%), and 720 (14.4%), respectively. Table 1 presents descriptive statistics by the level of life satisfaction for the 5000 participants nationwide.

**Table 1:** Summary of the questionnaire survey by the level of life satisfaction.

Variables	Life satisfaction				
	1, N = 381^a^	2, N = 834^a^	3, N = 1,011^a^	4, N = 2,250^a^	5, N = 524^a^
Age (years)	43.3 (14.2)	45.4 (15.1)	48.2 (15.5)	50.6 (16.7)	49.6 (17.6)
Sex					
Male	211/381 (55%)	401/834 (48%)	542/1,011 (54%)	1,088/2,250 (48%)	243/524 (46%)
Female	170/381 (45%)	433/834 (52%)	469/1,011 (46%)	1,162/2,250 (52%)	281/524 (54%)
Weight	61.6 (13.8)	60.1 (12.7)	61.0 (13.3)	60.2 (12.5)	59.6 (12.0)
Height	165.0 (8.9)	163.8 (8.8)	164.6 (9.0)	163.8 (8.9)	163.6 (8.4)
BMI	22.5 (4.0)	22.3 (3.7)	22.3 (3.6)	22.3 (3.4)	22.1 (3.3)

a Mean (SD); n/N (%).

### 3.2 Mental health and hot spring spa bathing habits

**Fig. 2:**
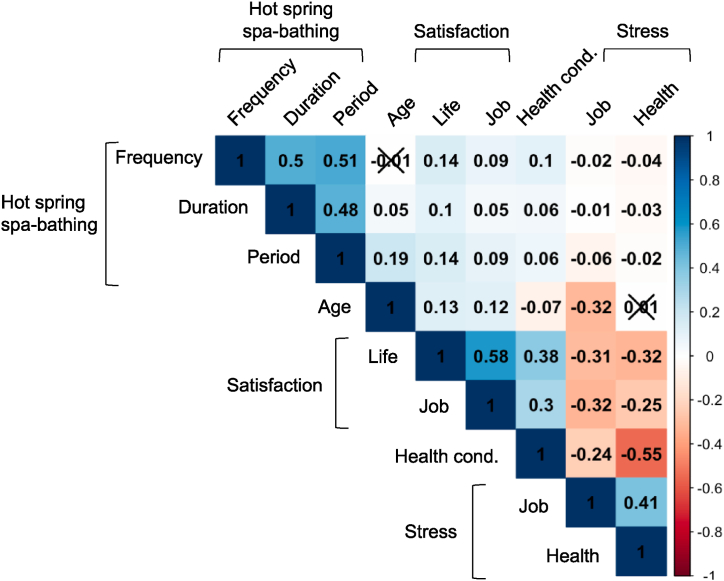
**Correlation plot between hot spring spa-bathing behavior and mental health indicators.** Each number indicates a Spearman's correlation coefficient. ′ × ′ indicates no significance (p = 0.05, n = 5000). Health cond. indicates 'subjective health conditions'.

To understand the effects of spa-bathing habits on mental health, we generated a correlation plot to analyze the relationship of hot spa-bathing behavior with each type of satisfaction and stress level (Fig. 2). Spearman's rank correlation was used to estimate the correlation coefficient. The results showed that hot spa-bathing behavior was significantly positively correlated with all satisfaction levels, whereas stress was significantly negatively correlated.

Regarding the correlation with hot spa-bathing frequency, the coefficient of life satisfaction was 0.14, job satisfaction was 0.09, subjective health was 0.10, job stress was −0.02, and health stress was −0.04, and all were significant. In addition, the duration of spa bathing had similar correlation coefficients, while the duration of bathing tended to be lower. Considering that the influence of factors other than hot spa bathing on satisfaction and stress is far more considerable, it can be assumed that hot spa-bathing behavior has a positive effect on mental health, even if the correlation coefficients with hot spa-bathing behavior ranged from 0.05-0–0.14 and −0.01-0–0.06.

The frequency, duration, and period of hot spa bathing were moderately positively correlated, with coefficients ranging from 0.48 to 0.51. Life satisfaction, job satisfaction, and subjective health conditions had weak to moderate positive correlation coefficients between 0.3 and 0.58. Stress at work and stress due to health status had a moderate positive correlation, with a coefficient of 0.41.

A possible trend of a positive correlation between age and satisfaction and life and work and a negative correlation between age and health satisfaction was observed. Compared to these correlation coefficients, the effect of hot spa-bathing behavior was considered large enough. These findings suggest that hot spa-bathing behavior may have a positive impact on mental health.

Further analysis was conducted for life satisfaction, which had the highest correlation coefficient with hot spa-bathing behavior. We examined the influence of hot spa-bathing behavior on the level of life satisfaction. Ordinal logistic regression was conducted using the frequency, duration, and period of hot spa bathing as explanatory variables, and all explanatory variables showed significance to life satisfaction (Table 2). All the variance inflation factor (VIF) values were sufficiently small (<10), which also indicated that there was no multicollinearity.

**Table 2:** The result of ordinal logistic regression of hot spring spa bathing habits on life satisfaction.

Variables	Life satisfaction		
	Coefficient	Pr(>|z|)	Standard Error
Spa-bathing frequency	0.0550 ***	0.0004	0.0157
Spa bath immersion duration	0.0511 *	0.0419	0.0251
Spa-bathing period	0.1267 ***	0.0000	0.0201

***p < 0.001, **p < 0.01, *p < 0.05.

### 3.3 Hot spring spa bathing habits and lifestyle trends

As a next step, we attempted to understand the relationship with hot spa-bathing behavior, mental health, and lifestyle. Exploratory factor analysis was used to extract the factor structure without prior assumptions. The overall Kaiser–Meyer–Olkin (KMO) test measure of sampling adequacy (MSA) was 0.77, which indicated that it was an appropriate method. We extracted factors with minimum residuals and rotated them with oblimin methods. The number of factors was decided by the maximum a posteriori probability (MAP) estimate, and a threshold of 0.30 loading was used to conclude when a component belonged to the factor. The factor analysis allowed us to decompose the results into four factors: mental status, health consciousness, diseases, and spa-bathing habits (Table 3). All the factor loadings for each component to each factor are indicated in Table 3. The factor correlations showed that mental status tended to be better with health consciousness, worse with illness, and good with hot spa-bathing habits (Table 4). For those individuals with a hot spa-bathing habit, the results showed that they tended to be more health-conscious and have better mental status and more diseases (Table 4). The result that peoples with higher hot spring bathing habits have more illnesses appears to be inconsistent with the conclusion that positive mental effects exist. Regarding this inconsistency, the positive correlation between mental status and hot spring habit (Table 4. Factor 1 and Factor 4) suggests that the positive mental effects may outweigh the negative effects of illness on mental health. In addition, hot springs have been used as balneotherapy to treat illness for centuries, so the results also reflect that people with illnesses frequently go to hot springs to improve their symptoms. These results suggest that spa-bathing habits may positively affect mental health, and individuals with diseases tend to use spa bathing to alleviate or improve symptoms.

**Table 3:** **Factor analysis of hot spring spa bathing behavior** Factor loadings above 0.30 are bolded.

	Factor 1	Factor 2	Factor 3	Factor 4
	Mental status	Health consciousness	Diseases	Spa-bathing habits
Life satisfaction	**0.6394**	0.0630	0.0673	0.0723
Job satisfaction	**0.6205**	0.0122	0.0849	0.0148
Subjective health conditions	**0.5730**	0.0226	−0.2067	0.0099
Job stress	**−0.5897**	−0.0602	−0.1326	0.0425
Health stress	**−0.6890**	0.0738	0.0956	0.0291
Free days	0.1081	0.1314	0.1407	0.0129
Breakfast	0.0714	**0.4277**	0.0237	0.0363
Vegetable intake	0.0743	**0.6690**	−0.0152	0.0765
Salt intake	−0.0177	**0.7778**	0.0392	−0.0357
Meat intake	−0.0286	**0.7434**	0.0298	−0.0330
Amount of food	0.0588	**0.5244**	−0.0532	−0.0089
Walking	−0.0407	**0.5110**	−0.0983	0.0360
Stairs	−0.0332	**0.4916**	−0.1066	0.0162
Smoking	−0.0336	−0.1954	0.0548	0.1342
Alcohol	0.0584	−0.0600	0.0542	0.2475
Allergies	−0.1420	0.0014	0.1102	0.0875
Apoplexy	0.0304	−0.0373	**0.4992**	−0.0362
Arrhythmia	−0.0470	0.0361	**0.3170**	0.0479
Asthma	−0.0822	−0.0055	0.1988	0.0456
Cancer	0.0003	0.0209	0.2940	0.0325
Chronic hepatitis	−0.0063	−0.0147	**0.4419**	−0.0337
Collagen disease	−0.0232	−0.0307	**0.3851**	−0.0385
Depression	−0.2204	−0.0113	0.1783	0.0147
DM	−0.0279	0.0002	**0.3723**	0.0209
Gout	−0.0256	−0.0379	0.2993	0.1071
Hyperlipidemia	−0.0284	0.0666	**0.3030**	0.1035
Hypertension	0.0133	0.0493	**0.3905**	0.1045
IHD	−0.0071	−0.0088	**0.4700**	0.0048
Renal disease	0.0121	−0.0163	**0.3364**	−0.0346
Spa-bathing frequency	0.0317	−0.0514	0.0365	**0.5888**
Spa bath immersion duration	−0.0252	−0.0153	−0.0470	**0.6674**
Spa-bathing period	0.0041	0.0470	0.0221	**0.6747**

**Table 4:** Factor correlation of the four factors.

	Factor 1	Factor 2	Factor 3	Factor 4
	Mental status	Health consciousness	Diseases	Spa-bathing habits
Factor 1	1	0.276	−0.124	0.159
Factor 2	0.276	1	0.041	0.146
Factor 3	−0.124	0.041	1	0.098
Factor 4	0.159	0.146	0.098	1

## 4 Discussion

This study showed that hot spring spa-bathing behavior has a positive association with mental health and the subjective health conditions in Japan. Regarding mental health, an increase in hot spa-bathing frequency showed a significantly positive correlation for all satisfaction levels and a negative correlation for all stress levels. In addition, life satisfaction was significantly affected by hot spa-bathing behavior. Hot spa-bathing balneotherapy has been reported to alleviate mental stress [10], and we also clearly demonstrated that individuals with hot spa-bathing habits tended to have lower mental stress. Other possible reasons for the positive mental effects are the relaxing effect of the opportunity to go outside, exposure to nature, and moderate exercise in nature. Indeed, Japan has many volcanic hot springs [2], so they are primarily located in the mountains. The relaxing effect of traveling and the proximity of the natural environment were previously reported to relieve mental stress [17,18]. Increased physical activity due to travel may also be a contributing factor. Some reports have indicated that exercise improves stress and psychological functions [19,20]. Subjective mental status was associated with higher health consciousness, increased hot spa-bathing behavior, and fewer illnesses, while hot spa-bathing behavior was also associated with more illnesses. A report described that most patients suffering from chronic pain believe that hot springs have a healing effect to improve their pain [21]. Therefore, our result may be due to the use of hot spa bathing to alleviate or ameliorate symptoms in the population with diseases.

Previous studies have reported that hot spring bathing is related to a reduced prevalence of hypertension [22]. In addition, it is known that psychosocial stress leads to the development of hypertension [23,24]. From this background, it is possible that the reduction of stress, as shown in this study, may contribute to the effect on hypertension. Since this is a cross-sectional observational study, we believe prospective follow-up and intervention studies are needed to estimate causal relationships.

The first limitation of this study is the low response rate which comes from the collection method. Rakuten Insight sent survey invitation emails to their monitors and asked them to respond to the survey voluntarily, so there is no compulsion to respond. Therefore, selection bias may have occurred, and the population is not fully representative of Japan. The second limitation is that because this is a cross-sectional study, it is possible that reverse causality may have occurred. Further research is needed to elucidate the psychological effects of hot springs through future follow-up and intervention studies. Also, the questionnaire was collected before the COVID-19 epidemic, and it is possible that the trend may have changed in Japan today. At the same time, however, we believe this study is precious for understanding the relationship between psychological status and hot spa bathing in the absence of the COVID epidemic. We also consider it important to follow up as part of future research to determine what changes in the mental effects of hot spa bathing have resulted from the COVID-19 epidemic. Another limitation is that income, which may be associated with hot spring use, has not been investigated in this study. Hence, future studies need to examine this possible confounding factor. The mechanism of both mental and physical health benefits of hot spring spa bathing has been largely unexamined, and little scientific evidence or consensus has been reached. This observational study suggested phenotypes and will provide a foundation for future clinical, medical, and biochemical research. Further research is needed to elucidate causal relationships and direct effects.

## 5 Conclusion

While reports of hot spring bathing improving stress have been found, there have been very few research reports on its effect on satisfaction. This study showed that the hot spring bathing habit is negatively correlated with stress and positively correlated with satisfaction in Japan. It also showed associations with a wide range of factors, including disease incidence and lifestyle habits, suggesting that bathing in hot springs may positively affect mental health rather than negative mental effects caused by illness. The results of this survey could support the mental health-promoting effects of hot spa bathing. Our results suggest that hot spring spa-bathing habits may improve satisfaction and stress. From our observations, it is necessary to analyze various environmental factors, such as exercise and diet, in addition to medical aspects for future research, considering that the effects of hot spa bathing are a combined effect that includes social behavior.

## Author contribution statement

Midori Takeda: Conceived and designed the experiments; Analyzed and interpreted the data; Wrote the paper.

Hiroki Nakamura: Conceived and designed the experiments; Analyzed and interpreted the data; Contributed reagents, materials, analysis tools or data; Wrote the paper.

Hajime Otsu; Koshi Mimori; Toyoki Maeda: Conceived and designed the experiments; Contributed reagents, materials, analysis tools or data.

Shunsuke Managi: Conceived and designed the experiments; Analyzed and interpreted the data.

## Data availability statement

Data will be made available on request.

## Acknowledgements and funding information

The authors are grateful for the helpful comments and suggestions of Shutaro Takeda from Kyushu University and the seminar participants at the IATSS, project number 2201A. This work was supported by JSPS KAKENHI, Grant Number JP20H00648. Any opinions, findings, and conclusions expressed in this paper are those of the authors and do not necessarily reflect the views of the abovementioned agencies.

## Declaration of competing interest

The authors declare that they have no known competing financial interests or personal relationships that could have appeared to influence the work reported in this paper.
