# Supplementary material for: Hot spring bathing practices have a positive effect on mental health in Japan
Source: Heliyon. 2023 Aug 30;9(9):e19631. doi: 10.1016/j.heliyon.2023.e19631 (PMC10558852; doi:10.1016/j.heliyon.2023.e19631)
Supplement: Multimedia component 2 [file mmc2.pdf]

An online questionnaire survey regarding health and hot spring spa bathing habit in Japan

Q1. Gender (1) Male (2) Female

Q2. Age (years)

Q3. Height (cm)

Q4. Weight (kg)

Q5. How often do you go to hot springs for spa bathing?

(0) Never been (1) Have not been for over a year (2) Approximately once a year

(3) Approximately once every six months (4) Approximately once every 3 months

(5) Approximately once a month (6) Approximately once every two weeks

(7) Approximately once a week (8) Twice a week or more (9) At least once a day

Q6. How long do you spend in the hot springs? (Total daily time in the bathtub)

(1) 10 min or less (2) 11-20 min (3) 21-30 min (4) More than 31 min (5) Unknown

Q7. How long have you been using the hot springs? (Lifetime to date)

(1) Less than 10 y (2) 10-19 y (3) 20-29 y (4) 30-39 y (5) More than 40 y (6) Unknown

Q8. Life satisfaction: How satisfied are you with your life overall? Choose (1) – (5) below.

Q9. Job satisfaction: Are you satisfied with your current job (working style)? Choose (1) – (5) below.

(1) Not satisfied (2) Mildly unsatisfied (3) Cannot say (4) Somewhat satisfied (5) Satisfied

Q10. Subjective health condition: Please choose the one that applies to your health condition.

(1) Poor (2) Mostly poor (3) Cannot say (4) Mostly good (5) Good

Q11. Job stress: What is your stress level (frustration or dissatisfaction) about your work or study? Choose (1) – (7) below.

Q12. Health stress: What is your stress level (frustration or dissatisfaction) about your illness or health problems? Choose (1) – (7) below.

(1) Do not feel stress at all

(2) Feel mild stress (short term: several days)

(3) Feel mild stress (medium term: weeks to months)

(4) Feel mild stress (long term: several years)

(5) Feel strong stress (short term: several days)

(6) Feel strong stress (medium term: weeks to months)

(7) Feel strong stress (long term: several years)

Q13. Please select any of the following diseases that you have had in the past.

Allergies Apoplexy Arrhythmia Asthma Cancer Chronic hepatitis Collagen disease Depression DM Gout Hyperlipidemia Hypertension IHD Renal disease

Q14. Do you smoke cigarettes?

(1) Never (2) Ex-smoker (3) Current smoker

Q15. How often do you drink alcoholic beverages?

(1) Never (2) Rarely (3) 1-2 days a week (4) 3-4 days a week (5) 5-6 days a week (6) Every day

Q16. How many holidays can you spend freely in a month on average? (days)

Please select options (1) – (5) for the following questions.

(1) Disagree (2) Somewhat disagree (3) Cannot say (4) Somewhat agree (5) Agree

Q17. Do you have breakfast every day?
Q18. Do you usually eat many vegetables?
Q19. Do you usually refrain from having too much salt?
Q20. Do you usually avoid eating too many animals' fat-rich foods?
Q21. Do you maintain a diet that allows you to maintain a proper weight?
Q22. Do you try to walk as much as possible without using a car?
Q23. Do you try to use the stairs as much as possible without using the elevator or escalator?
